# Cyclic combing of untreated and bleached human hair: Analysis of the time‐dependent breakage of hair through recording the formation of fibre fragments

**DOI:** 10.1111/ics.70016

**Published:** 2025-09-10

**Authors:** Thomas Davies, Gabriele Wortmann, Franz J. Wortmann

**Affiliations:** ^1^ Bossa Nova Vision/Dia‐Stron Inc Los Angeles California USA; ^2^ Department of Materials, School of Natural Sciences The University of Manchester Manchester UK; ^3^ F & GW – Consultants Aachen Germany

**Keywords:** claim substantiation for hair ‘strength’, combing, fibre breakage, hair treatment, kinetic analysis of fragment formation, statistics

## Abstract

**Objectives:**

Machine‐based cyclic combing of hair tresses under dry conditions is a proven method for evaluating hair strength and the impact of treatments. Recent advancements in image analysis allow for a detailed review of hair fragment lengths and quantities produced after specific combing cycles. Our aim is to provide an in‐depth analysis of the kinetics of hair fragment formation.

**Methods:**

We analysed the combing performance of untreated and bleached European straight hair, assessing two conditioning treatments. Hair tresses underwent 5000 combing cycles, with the increasing number of fragments recorded. Results were fitted using a three‐parameter Voigt–Kelvin model.

**Results:**

The fragment counts were log‐normally distributed in all cases. The model uses ln(*N*_0_) to indicate early fragment release, showing higher numbers for bleached hair compared to untreated hair, which significantly drop with conditioner treatments. The ln(*N*_∞_) parameter estimates the maximum expected fragments. The Failure Cycle Index (FCI) remains largely unchanged across materials according to its 95% confidence limits.

**Conclusions:**

The selected function's characteristics and the invariance of FCI indicate that friction primarily controls fragment formation in straight hair during combing. This suggest that there is no direct link between combing performance and tensile fatigue failure for this hair type. In contrast, textured hair will likely show more complex combing performance. Our analysis shows that the total number of fragments after many combing cycles can predict early failures and assess conditioning agents, thereby supporting product claims about ‘hair strength’.

## Graphical abstract

One important aspect of daily hair grooming routines is whether, after combing, conspicuous numbers of broken hairs are observed. Machine‐based cyclic combing of hair tresses serves as a good indicator for early hair breakage and as a suitable measure to determine the effect of an ingredient/product/process on hair ‘strength’.

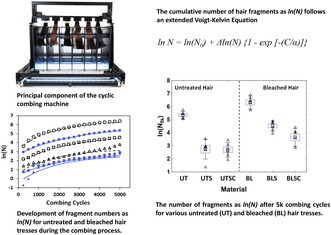

## Résumé

**Objectifs:**

Le brossage cyclique mécanique des mèches de cheveux à sec est une méthode éprouvée pour évaluer la résistance des cheveux et l’impact des traitements. Les progrès récents en matière d’analyse d’images permettent d’examiner en détail la longueur et la quantité des fragments de cheveux produits après des cycles de brossage spécifiques. Notre objectif est de fournir une analyse approfondie de la cinétique de la formation des fragments de cheveux.

**Méthodes:**

Nous avons analysé les performances de brossage des cheveux raides européens non traités et décolorés, en évaluant deux traitements après‐shampooing. Les mèches de cheveux ont été soumises à 5 000 cycles de brossage avec le nombre croissant de fragments enregistré. Les résultats ont fait l’objet d’un ajustement à l’aide d’un modèle de Kelvin‐Voigt à trois paramètres.

**Résultats:**

Le nombre de fragments était distribué selon une loi log‐normale dans tous les cas. Le modèle utilise ln(N0) pour indiquer la libération précoce des fragments, avec des valeurs plus élevées pour les cheveux décolorés que pour les cheveux non traités, qui diminuent de manière significative avec les traitements après‐shampooing. Le paramètre ln(N∞) estime le nombre maximal de fragments attendus. L’indice de cycle de défaillance (Failure Cycle Index, FCI) reste largement inchangé pour tous les matériaux selon ses limites de confiance à 95 %.

**Conclusions:**

Les caractéristiques de la fonction sélectionnée et l’invariance du FCI indiquent que la friction contrôle principalement la formation de fragments dans les cheveux raides lors du brossage. Cela suggère qu’il n’y a pas de lien direct entre les performances de brossage et la rupture par fatigue à la traction pour ce type de cheveux. En revanche, les cheveux texturés sont susceptibles de présenter des performances de brossage plus complexes. Notre analyse montre que le nombre total de fragments après de nombreux cycles de brossage permet de prédire les défaillances précoces et d’évaluer les produits après‐shampooing, ce qui corrobore les allégations des produits concernant la « résistance des cheveux ».

## INTRODUCTION

Combing and brushing are central parts of daily hair grooming processes. One important aspect of these hair care routines is whether after a relatively small number of strokes substantial numbers of broken hairs are found in the comb, brush or the sink. This may also be accompanied by the formation of split ends [1, 2, 3, 4]. If so, this will be a trigger of great concern for the consumer. To reduce the occurrence of hair breakage during grooming, a wide range of lubricating (conditioner) and ‘repair’ formulations is available to aid ease of combing (wet and dry) as well as to suppress the formation of broken hairs and split ends [2, 4].

To test the efficacy of such formulations in the laboratory, hand‐ and machine‐ combing for single tresses [1, 3, 5, 6] or cyclic combing for a group of tresses simultaneously [7, 8] are well established tools, namely under dry conditions. Apart from measuring combing forces [5, 9] one further approach is to collect the broken‐off fragments of hair after a given number of combing cycles and to determine their number or weight [3, 10, 11]. Robbins [3] associates the performance of hair in such tests specifically with ‘hair strength’. When comparing results with the reference hair material, a decrease in the numbers or weight of fragments would be associated with an improvement due to a treatment or product application. In addition, investigations have been conducted regarding the lengths of the released fibres [2, 11, 12].

Though the basic principles of machine‐based cyclic hair combing are well established [7], there have been significant developments in image capture and analysis. These developments enable users to simultaneously comb a number of tresses and to determine individually the number and the length of the hair fragments produced after defined cycles of combing [11].

Using such an appliance [11], this investigation focuses on analysing in detail the cumulative number of fragments during the ongoing combing process. The goal is to develop a meaningful analytical description of the kinetics involved. This approach will yield parameters that can be used to characterize fragment formation throughout the combing process.

Parameter values will be used to characterize untreated and bleached hair as reference materials, as well as to assess how fragment formation changes after different degrees of conditioning. The focus is on developing a detailed understanding of the nature of the parameters and their relationship to the practical aspects of hair combing.

## EXPERIMENTAL

This study examines the effects of cyclic combing on sets of both untreated and bleached hair tresses, without and with conditioner treatment. We determine and analyse the formation of hair fragments in relation to the number of combing cycles.

### Materials and treatments

Experiments were conducted on untreated (brown) (UT) as well as bleached (BL) straight European hair tresses (width: ≈25 cm; free length: ≈16 cm, weight: 2 g) purchased from International Hair Importers (Glendale, NY, USA).

A subset of six tresses of the UT‐group was washed with SLES (14%) [UT]. Additional sets were treated with a commercial ‘restoring shampoo’ [UTS], while another set received treatment with both the shampoo and a commercial conditioner [UTSC]. The active ingredients in both the shampoo and conditioner were silicones and oils. The products for the hair treatments were purchased from a common hair care range of the same company, chosen somewhat at random. Further specific details regarding the experiments are given elsewhere [11]. Equivalent treatments were applied to the BL‐group, resulting in the [BL]‐, [BLS] and [BLSC]‐sets of tresses. Before testing, tresses were gently combed out manually for five strokes on both sides of the tress, with a wide‐toothed comb, to remove any tangles that may have formed through the preparation process.

### Testing

For the experiments, we used the Sirtaki system with the 2.02 Software (Bossa Nova Vision, Los Angeles, CA, USA [13]). A set of six hair tresses was combed in parallel with four combs each along a circular path (16 cm) and the speed was set to 20 rpm, yielding a combing speed of about 220 mm/s. The imaging facility of the instrument allows detection of the number and lengths of hair fragments released from each hair tress after pre‐defined numbers of combing cycles (inspection cycles). For the current investigation, fragment numbers for each tress were automatically counted and analysed for length after every 250 cycles for a maximum of 5000 cycles. Fibre fragments shorter than 1 mm and longer than 16 cm were considered artefacts and removed from the data set. After detection and analysis, fragments are cleaned from the observation trays by a puff of air. The current analysis focusses on the cycle‐dependent kinetics of the development of fragment numbers.

Data analyses were conducted using Excel (Microsoft, 2016) and Statistica (V13, Tibco Software, 2017).

### Theory, model development and calculations

The combing process and the discontinuous image analysis provided for each tress (*i*) data for the number of hair fragments (*n*) with increasing combing cycles (*C*_*j*_), where the index *j* gives the running count of inspections. For the analysis of the data, we consider them in the form of the cumulative number of fragments for each tress (*N*_*i,j*_), given as:

(1) $$N_{i,j}=\sum_{j}n_{i,j}$$

**FIGURE 1:**
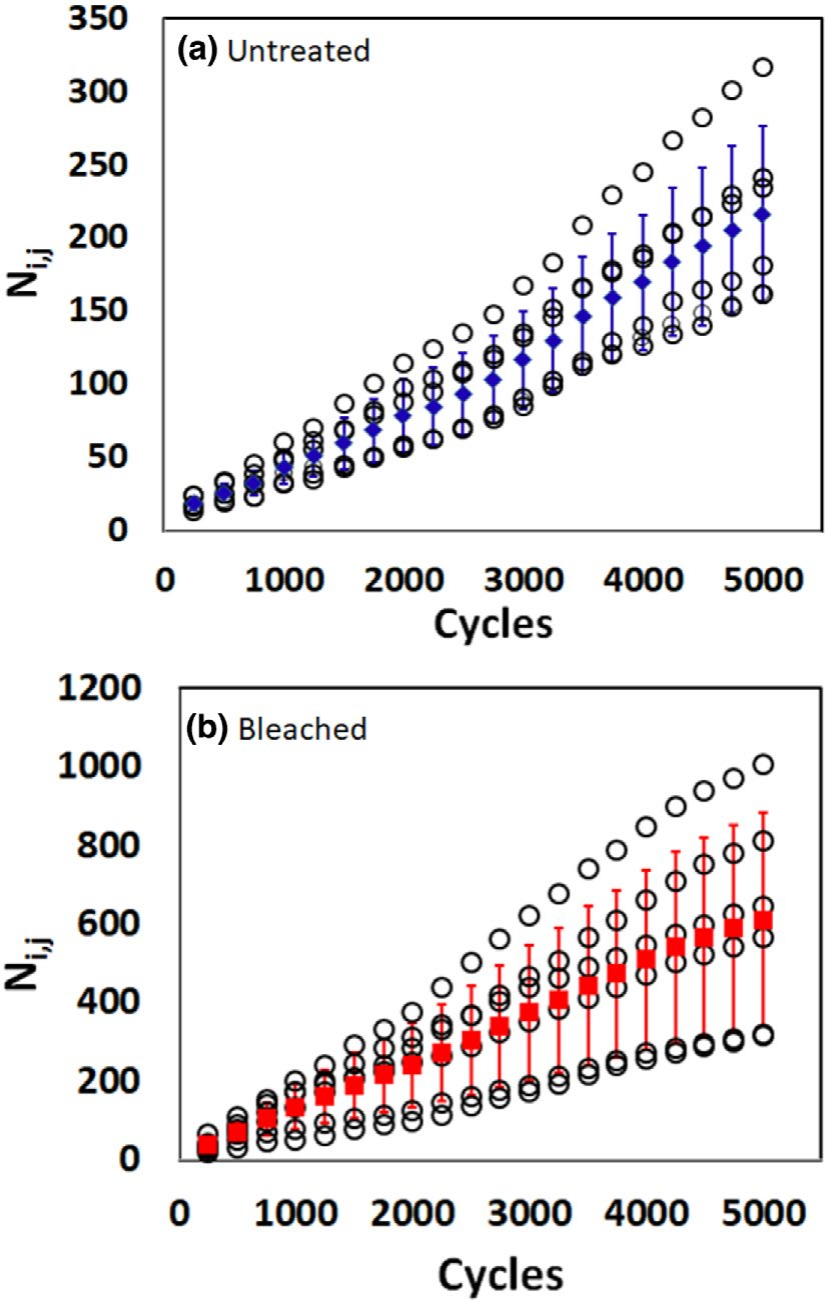
Cumulative number of fragments for each tress (*N*_*i,j*_) at each inspection cycle (*C*_*j*_). (a) Untreated, (b) Bleached hair. Individual values for each of the six tresses in a test are given (**○**). Markers and whiskers give the arithmetic means and standard deviations for untreated (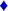) and bleached (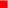) hair, respectively. Different *y*‐scales are applied in (a, b) to enable the qualitative comparison of data variance.

**FIGURE 2:**
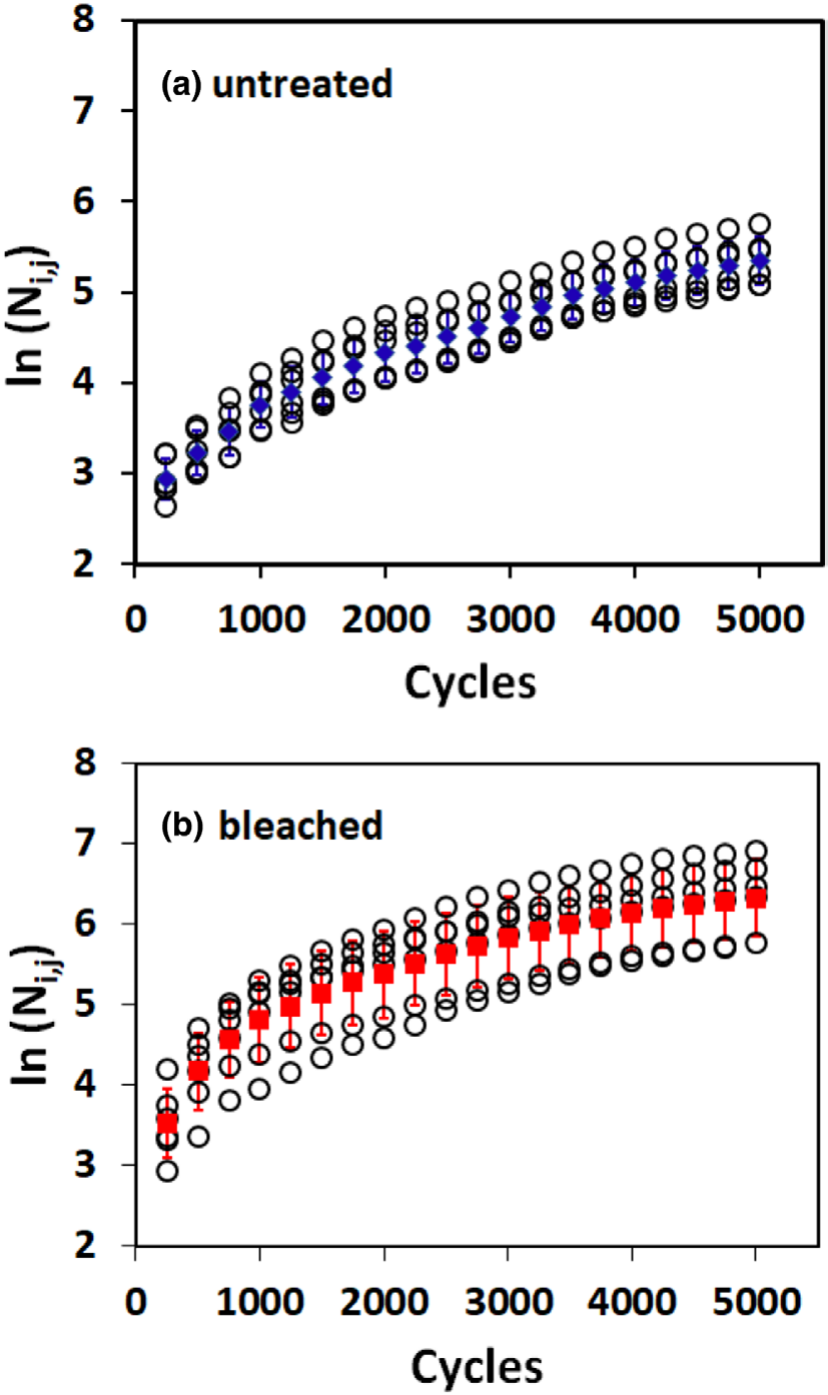
Cumulative number of fragments (**○**) as ln(*N*_*i,j*_) for each of the six tresses in a test and at each inspection cycle for untreated (a) and bleached (b) hair. Solid markers (untreated: 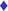; bleached: 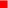) and whiskers give arithmetic means and standard deviations (see Table S2).

As shown below, data for *N*_*i,j*_ are not normally but rather log‐normally distributed (see Figures 1 and 2). Figure 2 shows that the distribution of ln(*N*_*i,j*_)‐data for the whole testing process is quite homogeneous across the tresses for both untreated and bleached hair.

The course for both sets of ln(*N*)‐data (see Figure 2) suggests that they have a defined start [ln(*N*_0_)] and approach a final value [ln(*N*_∞_)]. *N*_0_ as such defines for each tress the limiting number of fragments, which are formally released at *C* = 0. This value will be close to that experimentally observed at the earliest stage of combing. The assumption of *N*_∞_ derives first from the course of the data in Figure 2 but also from the simple fact that each tress can only release a finite number of fragments. With the observation that ln(*N*_*i,j*_)‐data are log‐normally distributed, it is these data for which a model‐based analysis should be developed.

This approach would work for the current data sets for UT‐ and BL‐tresses. Here, we consistently found for the initial number of fragments *n*_*i*,1_ ≥ 1. However, the situation is different for conditioned UT‐ and BL‐samples, where *n*_*i*,1_ = 0 may occur, so that ln(*n*_*i*,1_) becomes undefined.

To enable, against this background, to pursue our analysis of the cumulative fragment data for all samples, we found it at this stage feasible to base our consideration on the sum of *N*_*i,j*_ across all tresses according to:

(2) $$N_{j}=\frac{\sum_{i=1}^{k}N_{i,j}}{k}$$

where *k* = 6 is the number of tresses tested simultaneously for the current experiments. We used the results for ln(*N*_*j*_) for the development of a model‐based analysis.

There are numerous potentially feasible approaches to model the type of curve defined by the data in Figure 2. On the basis of our experience [14], we found that the equation for the Voigt–Kelvin model [15] is well suited to describe how ln(*N*_*j*_)‐data change for the equally spaced combing cycles (*C*_*j*_).

We aim to describe the cumulative number of fragments *N* observed for a set of tresses over the range of observation cycles (*C*) (see Figure 2) generally as:

(3) $$\ln N=\ln\left(N_{0}\right)+∆\ln\left(N\right)\left\{1-\exp\left[-{\left(C/\alpha \right)}^{\beta }\right]\right\}$$

where *C* is the number of combing cycles, α the *specific failure cycle* and β the *shape factor*. Pre‐trials with the data sets showed us that they were all well described by applying a shape factor of β = 1. Through this, the right‐hand side of Equation (3) reverts to the classical Voigt–Kelvin form [15].

Applying:

(4) $$∆\ln\left(N\right)=\ln\left(N_{\infty }\right)-\ln\left(N_{0}\right)$$

Equation (3) yields with β = 1:

(5) $$\ln N=\ln\left(N_{\infty }\right)-\left[\ln\left(N_{\infty }\right)-\ln\left(N_{0}\right)\right]\exp\left[-\left(C/\alpha \right)\right]$$

Given the suggested connection between fragment number released by combing and hair fatigue failure performance [7, 16], it is of interest to note that the exponential expression on the right‐hand side of Equation (5) is consistent with a cumulative Weibull distribution (CWD) function. A shape factor of unity suggests that the rate of fragment formation is constant throughout the process.

Equation (5) is used to fit the cumulative fragmentation data for all samples. Given the exponential nature of Equation (5), we perform the fit on the ln(*C*) scale, obtaining values for ln(*N*_0_), ln(*N*_∞_) and ln(α). We refer to the latter variable as *failure cycle index (FCI)*, in analogy to a previous investigation [17]. The fitting process is carried out using the Non‐linear Estimation tool available in Statistica.

## RESULTS

Figure 1 summarizes for untreated and bleached hair the cumulative number of fragments obtained from each of the six tresses (*N*_*i,j*_) versus the number of combing cycles (*C*_*j*_). The respective means across all six tresses together with their standard deviations (whiskers) are also shown. The mean values increase for both types of samples systematically with *C*_*j*_. BL‐samples show, as to be expected [7, 16, 18], much higher fragment numbers than UT‐material. To provide data sets of similar appearance, the *y*‐scales for Figure 1a,b are adjusted accordingly.

To detail the basis of Figure 1, Table S1 summarizes the means of the cumulative fragment numbers for each inspection count for untreated and bleached materials along with their standard deviation (STD) and the coefficients of variation (CV) across the six tresses. Means for UT hair are consistently about a factor of three lower than for BL hair.

In contrast to literature data [7], it is noteworthy that the standard deviations increase systematically with increasing *C*_*j*_‐values, while CV‐values remain relatively constant (see Table 1). CV is substantially lower for untreated (28%) compared to bleached hair (44%). This observation of largely constant CV‐values supports the hypothesis [11] that *N*_*i,j*_‐data across the tresses follow a log‐normal rather than a normal distribution.

**TABLE 1:** Values for ln(*N*_*0*_) and ln(*N*_∞_) (see Equation 5) together with their 95%‐confidence range as determined by the non‐linear fit of ln(*N*_*j*_) data for each set of six tresses.

Sample	ln(*N*_0_) ± *q*_95%_	*N*_0_ range	ln(*N*_∞_) ± *q*_95%_	*N*_∞_ range	ln(α) ± *q*_95%_	α [cycles] range	*R* ^2^
UT	2.75 ± 0.19	19–13	5.89 ± 0.45	567–230	7.97 ± 0.32	3984–2100	0.883
UTS	*0.43* ± *0.46*	2–0	2.89 ± 0.72	37–9	7.78 ± 0.75	5064–1130	0.502
UTSC	*−0.40* ± *0.73*	1–0	2.62 ± 0.56	24–8	7.53 ± 0.62	3463–1002	0.508
BL	3.33 ± 0.42	43–18	6.44 ± 0.35	889–441	7.47 ± 0.37	2540–1212	0.721
BLS	1.77 ± 0.26	8–5	4.87 ± 0.40	194–87	7.78 ± 0.34	3361–1703	0.832
BLSC	0.64 ± 0.34	3–1	3.31 ± 0.19	33–23	7.5	1808	0.512

*Note*: ln(*N*_0_)‐values, which are not significantly different from zero at the 95%‐level are given in italics. For each variable, values for the upper and lower limit of the 95%‐confidence range are given for the linear scale. *R* ^2^ is the coefficient of determination as a measure of the goodness of fit. The restricted value of the *FCI* = ln(*α*) for sample BLSC is marked (see text).

Figure 2a,b summarize the data for ln(*N*_*i,j*_) for untreated and bleached hair, similar to Figure 1a,b. These graphs are possible for these two samples due to the absence of zero‐counts for *N*_*j*_ at early stages of combing. The graphs clearly show the consistency of the standard deviation across all inspection cycles, supporting the assumption of a log‐normal distribution for the *N*_*i,j*_ data across the tresses. Table S2 summarizes the means at each inspection count for untreated and bleached materials along with their standard deviation (*STD*) and the coefficients of variation (CV) across the six tresses.

The curves illustrate a non‐linear exponential relationship that has both a lower and an upper limit, thereby validating the use of Equation (5). The data presented in Figure 2 (see also Table S2) confirm the assumption of satisfactory homogeneity across the tresses. On this basis, all data from a specific set of tresses for a particular material were combined according to Equation (2) and fitted using Equation (5).

**FIGURE 3:**
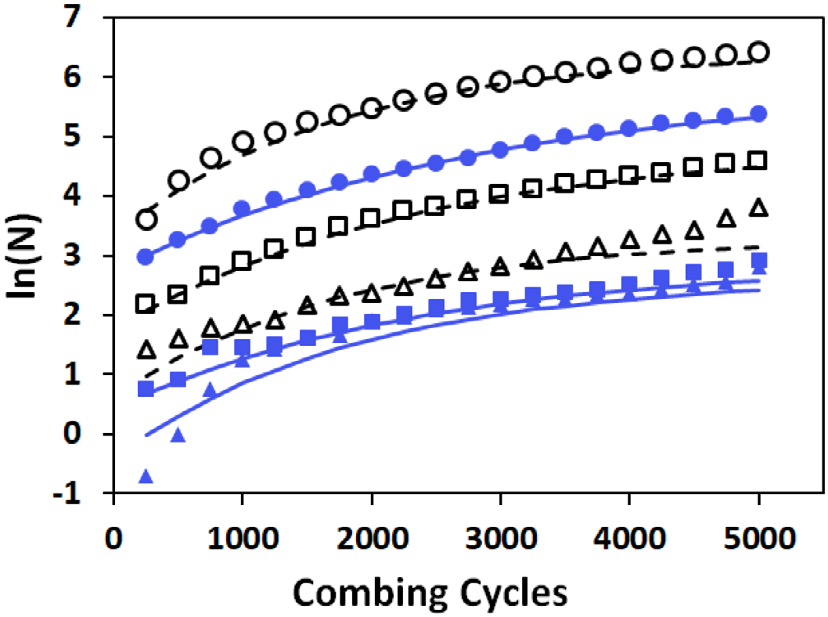
ln(*N*_*j*_) results for all samples. UT: 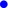, UTS: 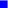, UTSC: 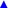; BL: օ, BLS: **□**, BLSC: Δ. Lines through the data points are fits according to Equation (5) with the variable values in Table 1. Solid lines are applied for UT‐ and broken lines for BL‐samples, respectively.

Figure 3 summarize the ln(*N*_*j*_)‐data for all samples and the fits according to Equation (5), using the variable values as detailed in Table 1.

Table 1 summarizes the results of the fits of the ln(*N*_*j*_)‐data across all samples. For all materials, the variable values (see Equation 5) are statistically significant, except for BLSC. In this case, the very small number of fragments only supports two significant variables. In view of the observation that the *FCI* is essentially constant across samples from UT to BLS, we chose a constant value of *FCI* = 7.5 for the BLSC fit. We consider this approach as practically feasible at this early stage of our investigation. However, we will further evaluate the necessity of restrictions of the fits when more comprehensive data become available in the future. We consider it as worth noting that the data for untreated hair given by Park and Evans [7] are well fitted by Equation (5) with adequate agreement of the variable values.

## DISCUSSION

The results in Figure 1 show that cumulative fragment numbers, in all cases, increase with the number of combing strokes up to the chosen experimental limit. The curve form is distinctly different from results reported in the literature [19]. The data are satisfactorily homogeneous across the tresses in a given experiment. Not unexpectedly [7, 16], fragment numbers are systematically higher for bleached compared to untreated hair, irrespective of the subsequent conditioner treatment. However, the systematic increase of the standard deviation with inspection cycles (see Figure 1a,b) leads to the conclusion that data for *N*_*i,j*_ are log‐normally rather than normally distributed. Accordingly, Figure 2a,b, show for ln(*N*_*i,j*_) an essentially constant level for the standard deviation against combing cycles. Standard deviations for ln(*N*_*i,j*_) are systematically higher for bleached compared to untreated hair.

Considering the course of the data with increasing combing cycles in Figure 2a,b, we developed Equation (5) to fit the data. The fits for all samples are shown in Figure 3. Equation (5) contains three parameters, namely, ln(*N*_*0*_), ln(*N*_*∞*_) and ln(α). *N*_*0*_ predicts the number of fragments, which are released upon the start of the combing process, while *N*_∞_ estimates the total number of fragments, which are expected to be eventually released by the experiment. *FCI* {ln(α)} characterizes the cycle‐dependence of the simple exponential process as fragment numbers develop from ln(*N*_*0*_) to ln(*N*_∞_). The results for all three parameters, along with suitable precision measures, are summarized in Table 1. To aid the discussion, both the logarithmic and linear values are provided.

**FIGURE 4:**
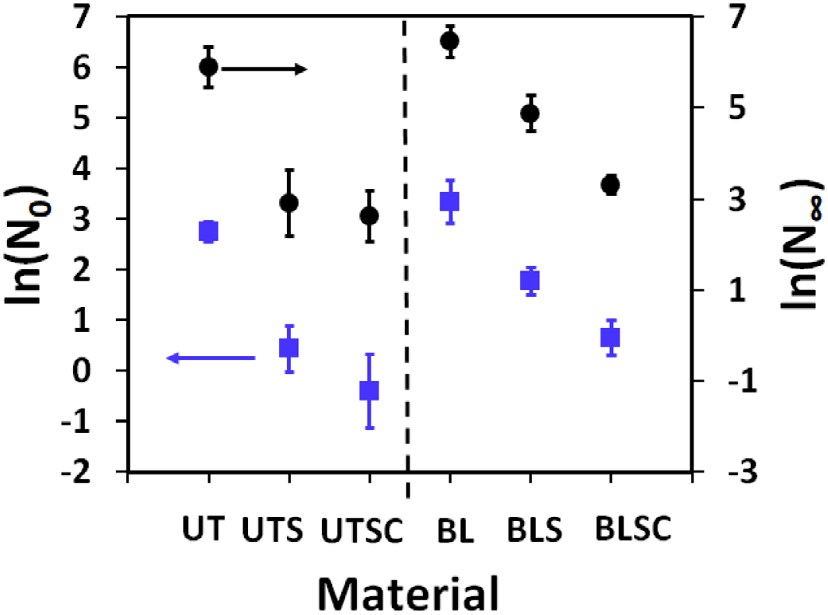
The initial and final number of hair fragments according to Equation (5) as ln(*N*_0_) (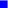) and ln(*N*_∞_) (●), respectively, for all untreated and bleached samples. Whiskers give the 95% confidence range.

We note from Table 1 that the coefficients of determination of the fits tend to drop significantly for conditioner‐treated hair (UTS, UTSC, BLSC: *R* ^2^≈0.5). This is attributed to the instability of the fit when fragment numbers, namely, during the early stages of cyclic combing, may be zero or very low. Figure 4 summarizes the results for ln(*N*_*0*_) and ln(*N*_∞_) for all samples.

Figure 4 shows that ln(*N*_0_) and ln(*N*_∞_) are consistently higher for bleached hair. Combing forces for straight European hair are largely controlled by the friction between hairs and comb [18]. This friction between comb and hair is higher for BL than for UT material [3, 18]. Higher friction forces as well as higher overall fragility [16] will lead to higher fragment numbers for BL hair. Conditioner treatments reduce friction [20]. Accordingly, fragment numbers decrease with the efficacy of lubrication. The lack of change from UTS to UTSC (see Table 1) just indicates that there is a limit for the lubrication/conditioner effect. From a practical perspective, it is interesting to note that ln(*N*_0_)‐ and ln(*N*_∞_)‐values for strong conditioner treated bleached hair (BLSC) fall into the same range as the related conditioned UT hair.

The differences for ln(*N*_0_) and ln(*N*_∞_) for the samples are already quite pronounced in most cases. However, the efficacy of the conditioner effects is especially apparent for the linear *N*_0_ and *N*_∞_ values (see Table 1). For UT‐material, we have seen a drop in the *N*_0_ range for untreated hair from 13 to 19 initial fragments to 0–1 after the intensive conditioner treatment (UTSC). For bleached hair, the initial *N*_0_ range drops from a high range of 18–43 (BL) to 1–3 after the same treatment (BLSC). Contrary to our expectations, we found that the reduction of hair fragments through the lubrication efficiency from the current conditioning treatments was more pronounced for UT‐ than for BL hair. However, for the current cases of a single bleach, the differences compared to untreated hair are not very large on the ln‐scale. We anticipate that these differences will become more pronounced with stronger or repeated bleaching treatments.

**FIGURE 5:**
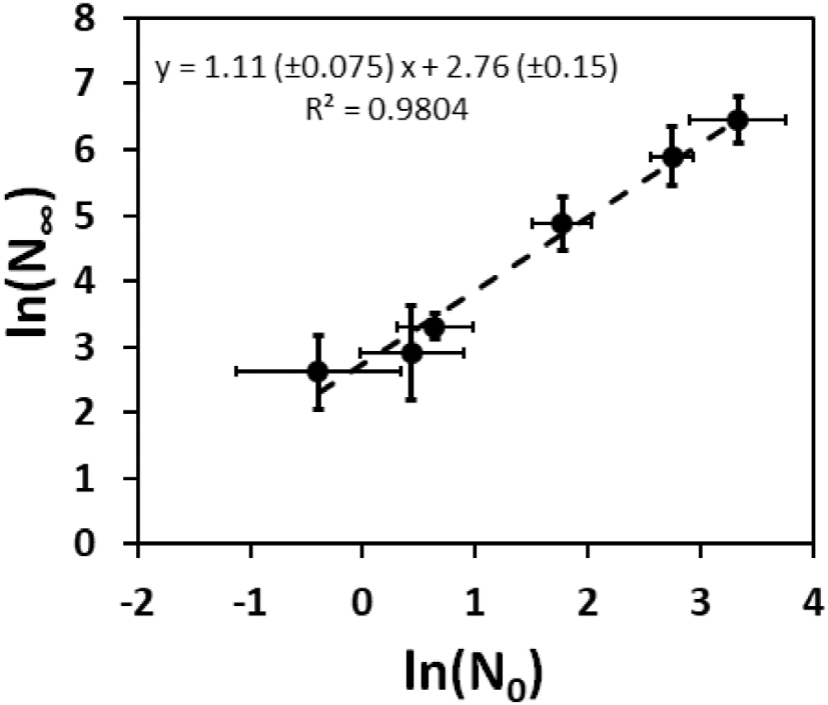
Plot of ln(*N*_∞_) versus ln(*N*_*0*_) for all samples (see Table 1). Whiskers give the 95%‐confidence limits for both parameters. The values (± standard error) for slope and intercept of the regression equation and the coefficient of determination (*R* ^2^) are given.

Figure 4 also shows that, ln(*N*_0_) and ln(*N*_∞_) show overall very similar changes with treatment, indicating a relation between the parameters. Figure 5 shows the plot of ln(*N*_∞_) versus ln(*N*_*0*_), confirming a strong correlation.

The correlation between the variables in Figure 5 is very strong (*R* ^2^ = 0.984). It is interesting to note that the 95%‐confidence range of the slope (sl) encloses the value of sl = 1 (0.90–1.33) and that the intercept (ic) has a value of ic = 2.8 (±0.43). This leads to:

(6) $$N_{\infty }\approx N_{0}\exp\left(2.8\right)$$

The correlation in Figure 5 shows that *N*_∞_ is essentially constant at *N*_∞_ ≈ 16 *N*_0_ across all samples. Without putting too much emphasis on the numerical factor in Figure 5 as such, the analysis nevertheless suggests a constant factor connecting *N*_0_ and *N*_∞_.

We found this result rather surprising. Against the background of previous research [1, 2, 12], we initially assumed that *N*_0_ and *N*_∞_ represent two different groups of fragments. *N*_0_ represents, for example the performance of pre‐damaged fibres, for which fragments are released very early in the combing process. In contrast, *N*_∞_ denotes the maximum number of fibres expected to be released along a different pathway during the entire combing process. However, Equation (6) shows that the end point of the combing process may in fact be predicted by its starting point, in that *N*_∞_ is a strict multiple of *N*_0_. In summary, this result does not support the assumption of different pathways of fragment formation during combing [1, 2].

**FIGURE 6:**
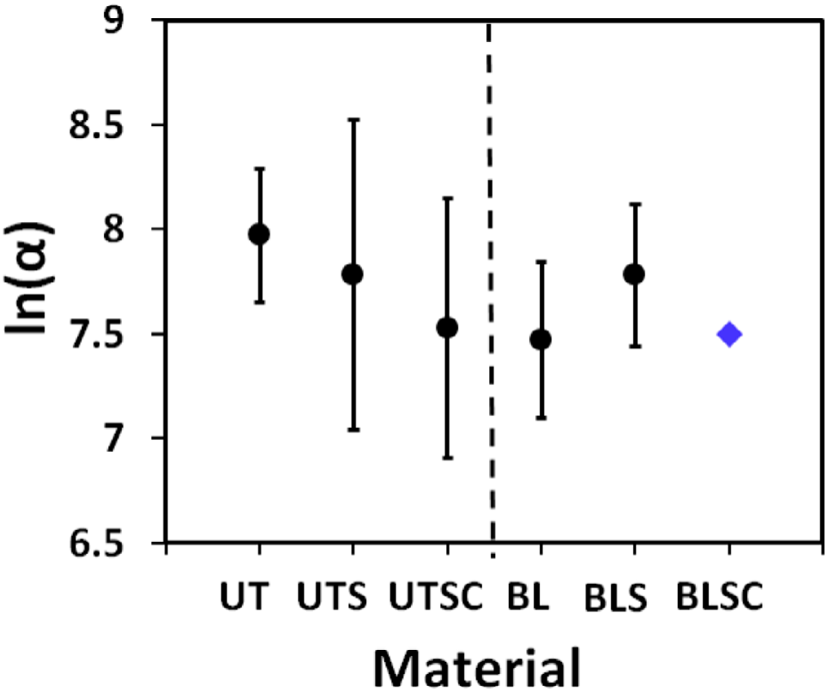
*FCI* {ln(α)} values from fitting the extended Voigt–Kelvin equation (Equation 5) to the experimental data. The whiskers give the individual 95%‐confidence limits. For the BLSC sample, the fixed value of ln(α) = 7.5 is given (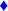).

One of the parameters in the extended Voigt–Kelvin equation (see Equation 5) to model the experimental data (see Figure 3) is the *FCI*. The index defines the position of the CWD‐function as part of the Voigt–Kelvin equation on the ln(*C*) scale and thus defines the cycle/time dependence of a fragmentation process. Using a defined shape of the CWD‐function (β = 1, see Equation 5), the process as such is material invariant. This assumption is supported by high coefficients of determination in Table 1, namely, for those cases where reasonably large numbers of fragments are observed. Figure 6 summarizes the results for *FCI* for all untreated and bleached samples.

The results in Figure 6 show that the 95%‐confidence limits of the *FCI* overlap. Without a further detailed analysis, we assume that ln(*a*)‐values are rather invariant around *FCI*≈7.5 across all samples. Though *N*_0_ and *N*_∞_ are generally different for the samples (see Table 1), the constancy of the shape as well as the location of the CWD function on the ln(*C*)‐scale imply that fragmentation follows in all cases essentially an invariant cycle/time law during the combing process. With β = 1, *FCI* does change neither with hair processing (UT vs. BL) nor with conditioner treatment (S & SC). Due to the log scale, this is only seemingly in contrast to the rather large range for individual values for the *specific failure cycle* (α) of 1–5k (see Table 1).

The invariance of the *FCI* leads to a further interesting aspect when comparing the UT‐ versus the BL‐sample. It has been suggested that there is a direct link between combing fragmentation‐ and tensile as well as fatigue failure–measurements [6, 7, 16, 21, 22]. We observed, however, that the shape of the CWD‐function is substantially broader for fatigue failure (β < 1) and *FCI* decreases by about 2.5 units between untreated and bleached hair. This amounts to a factor of about 10 [17]. In contrast, for combing the change of the *FCI* is less than 0.5 of a unit and inconsistent across UT‐ and BL‐samples (see Figure 6).

For our chosen experimental conditions, this leads us to suggest that, at least for straight hair, there is no simple link between fatigue failure‐ and the combing performance. It rather suggests that combing of straight hair is a purely friction controlled process. For this special case, our results support the scepticism of Kamath and Robbins [10] regarding the link between the methods. However, we would expect a somewhat different picture for textured hair, where the combing force curves show that, for example ‘snagging’ will introduce a tensile component to the fragmentation process. We expect that this tensile component will become increasingly dominant at higher degrees of hair curliness [3], for longer hair or with higher combing speeds, possibly leading to a more consistent correlation with fatigue failure experiments.

To model various aspects of fibre interactions during detangling by combing, which may lead to fibre splitting, Taylor et al. [4] devised a number of dynamic mechanical tests for fibre pairs. These tests, in our view, reflect well certain limiting geometries of hair tangles, as shown in various publications [1, 2, 3, 12, 23].

**FIGURE 7:**
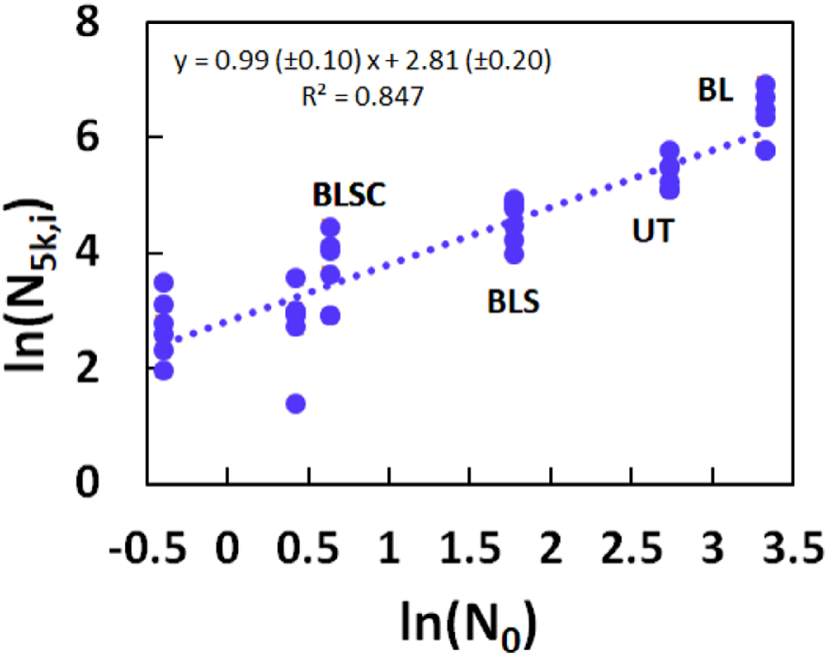
Plot of experimental data for ln(*N*_5k,*i*_), that is for each tress, vs ln(*N*_0_) for a set of tresses. The regression line (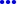) is shown. The regression equation together with the coefficient of determination (*R* ^2^) is given. For further details, see text.

Given the relationship between ln(*N*_0_) and ln(*N*_∞_) (see Figure 5), we expect and observe an excellent correlation between ln(*N*_*0*_) and ln(*N*_5k_), which is the experimental result for the cumulative number of fragments after 5000 combing cycles. This aspect of our investigation is summarized in Figure 7, where we plot for each tress ln(*N*_5k,i_) against ln(*N*_0_) for all materials.

**FIGURE 8:**
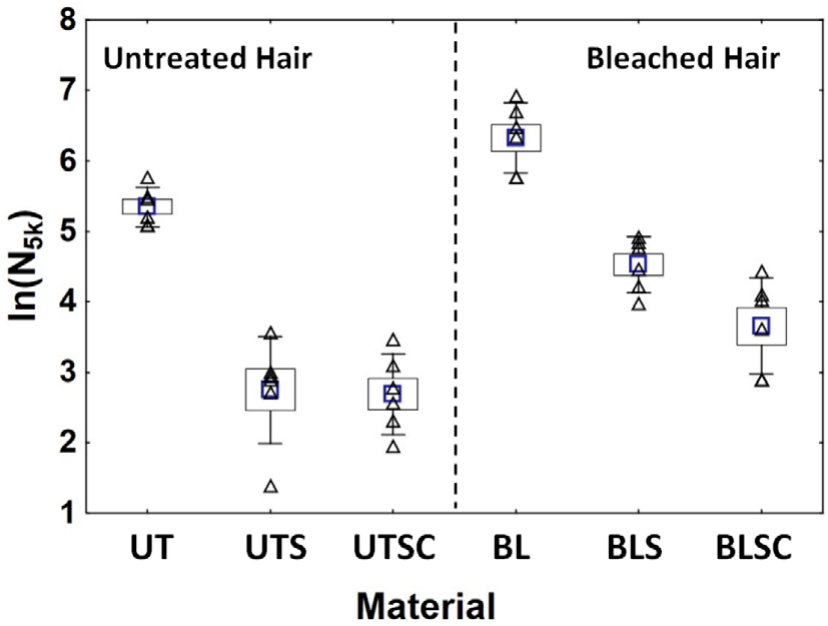
The cumulative fragment number after 5000 cycles (*N*_5k,*i*_) as ln(*N*_5k,*i*_) for the individual tresses of all materials. The individual values for each tress (Δ), their arithmetic mean (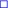), their standard error (SE: Box) and 95% confidence limits (whiskers) are given. Individual differences are significant between all data sets with the exception of UTS versus UTSC (*p* = 0.86), as determined by the non‐conservative LSD test (Statistica).

The fit of the data is, as expected, very similar to that in Figure 4 since ln(*N*_5k_) is already quite close to ln(*N*_∞_). We only observe slight numerical differences in slope and intercept, at a high coefficient of determination (*R* ^2^ = 0.847).

ln(*N*_5k_) is thus a variable to comprehensively describe the hair fragmentation process with the advantage of being readily experimentally available. Figure 8 summarizes the results for ln(*N*_5k,*i*_) for all samples, summarizing and condensing the results of Figure 7. In Figure 8, the differences between all samples are significant well beyond the 95%‐level with the exception of UTS versus UTSC (*p* = 0.86) (LSD‐test).

The results for *N*_5k_ or more specifically ln(*N*_5k_) show that hair breakage increases, as expected, through bleaching. The chemical damage is effectively mitigated through the conditioner by reducing the friction between hair and comb [24], thereby improving combing ease.

## CONCLUSIONS

Though results for hair fragment formation during cyclic combing appeared rather complex at first sight, we found that the data could be well systematized in a rather straightforward analytic structure. Cumulative fragment numbers turned out to be log‐normally distributed and to be well described by an extended Voigt–Kelvin equation (Equation 5) with combing cycle numbers.

The observation that one of the parameters of the model, namely, FCI {ln(α)} is essentially constant across all materials confirms, in our view, that combing induced fragmentation for straight hair is controlled by friction only. The dissimilarity between the parameter values for combing compared to fatigue failure leads us to suggest that there is no link between the methods for straight hair [17, 25].

The model also includes the parameter *N*_0_, which is a measure for the early release of fragments during combing. *N*_0_ is a critical metric; if its value is high, it would obviously be a potential trigger for serious consumer concerns in practice. However, at low combing cycle numbers, fragment numbers may be too small to measure reliably. It is thus the strict relationship between ln(*N*_0_) and ln(*N*_5k_) which allows the latter to be used as a reliable indicator for the resilience of hair against cyclic combing. This relationship also provides a basis for the comparison and differentiation of lubrication/conditioning agents, in close relation to the practical situation.

This observation may help resolve the longstanding debate within the hair cosmetic science community regarding the relevance of cyclic combing with a large number of combing strokes for real‐world use. In practice, consumers will typically use much fewer combing strokes. Our investigation shows that, at least for straight European hair, results from extensive combing (e.g. *N*_5k_) may serve as good predictors of hair fragment release during early grooming and thus of combing ease. This finding is expected to also apply to lower numbers of combing cycles, as long as enough fragments are produced to establish a cumulative curve that can be reliably fitted. Such experiments would be beneficial to increase sample throughput.

There are many types of combs and brushes available for hair grooming, and we anticipate each tool to yield different results. Nevertheless, considering the friction control for straight hair, we believe that a similar analysis approach may be applicable for combs and brushes, though specific variable values will differ.

However, we consider our considerations as valid for straight hair only, where we observe a strict coherence between parameters. We expect a distinctly different picture for curly hair, where with increasing texture, friction becomes an increasingly minor effect. Increasing combing forces are more controlled by tensile forces and impact induced by hair texture. For such hair, ‘snagging’ will occur with hairs looped over other hairs, leading to severe hair bending and localized impact stresses [1, 2, 4, 12, 23]. For such a multifactorial phenomenon [3], we may expect different and more complex kinetics of fragment formation.

Finally, we recognize that the term ‘strength’ has originally specifically been linked to hair performance during grooming (fragment formation and ‐length) [3]. However, because the term ‘strength’ is frequently used in the context of other tests (e.g. tensile‐, fatigue failure‐test) [21, 22] we consider the term as too ambiguous when applied to hair combing. Therefore, we propose using the term ‘grooming or combing resilience’ instead [11].

## CONFLICT OF INTEREST STATEMENT

TD is Business and Development Manager at Bossa Nova Vision. The company develops, builds and markets testing equipment, namely, for the hair cosmetic industry. FJW and GW are partners in F & GW – Consultants GbR. The company provides academic support for the hair cosmetics industry on a consultancy basis.

## Supporting information


Table S1:
Table S2:

## Data Availability

The data that support the findings of this study are available from the corresponding author upon reasonable request.
