# Supplementary material for: Cyclic combing of untreated and bleached human hair: Analysis of the time‐dependent breakage of hair through recording the formation of fibre fragments
Source: Int J Cosmet Sci. 2025 Sep 10;48(2):201–10. doi: 10.1111/ics.70016 (PMC13068040; doi:10.1111/ics.70016)
Supplement: Supplementary file 1 — Table S1: Table S2: [file ICS-48-201-s001.docx]

**Supplement**

**Table S1:**

Mean (*N_j_*) of the cumulative number of fragments for a set of six tresses for untreated (UT) and bleached (BL) hair. The standard deviations (STD) and the Coefficients of Variation (CV) are given.Number of tresses = 6.

| **Inspection Count** | **Cycles** | **UT**  **N_j_ ± STD** | **CV,%** | **Bleach**  **N_j_ ± STD** | **CV, %** |
| --- | --- | --- | --- | --- | --- |
| 1 | 250 | 19 ± 4.6 | 23.7 | 37 ± 17 | 45.3 |
| 2 | 500 | 26 ± 6.3 | 24.4 | 71 ± 29 | 41.3 |
| 3 | 750 | 33 ± 8.6 | 26.0 | 105 ± 42 | 40.1 |
| 4 | 1000 | 44 ± 11 | 25.3 | 135 ± 59 | 43.2 |
| 5 | 1250 | 51 ± 14 | 27.6 | 161 ± 68 | 42.1 |
| 6 | 1500 | 60 ± 18 | 30.0 | 190 ± 83 | 43.5 |
| 7 | 1750 | 69 ± 21 | 31.2 | 216 ± 95 | 44.0 |
| 8 | 2000 | 79 ± 25 | 31.5 | 241 ± 109 | 45.0 |
| 9 | 2250 | 85 ± 26 | 31.3 | 274 ± 123 | 45.1 |
| 10 | 2500 | 94 ± 28 | 29.6 | 305 ± 139 | 45.5 |
| 11 | 2750 | 104 ± 30 | 28.6 | 341 ± 156 | 45.8 |
| 12 | 3000 | 117 ± 34 | 28.8 | 375 ± 172 | 45.9 |
| 13 | 3250 | 131 ± 36 | 27.2 | 407 ± 184 | 45.2 |
| 14 | 3500 | 147 ± 40 | 27.1 | 443 ± 202 | 45.5 |
| 15 | 3750 | 160 ± 44 | 27.3 | 475 ± 211 | 44.6 |
| 16 | 4000 | 170 ± 46 | 26.9 | 511 ± 228 | 44.7 |
| 17 | 4250 | 184 ± 50 | 27.4 | 541 ± 244 | 45.1 |
| 18 | 4500 | 195 ± 54 | 27.8 | 566 ± 255 | 45.0 |
| 19 | 4750 | 206 ± 58 | 28.0 | 589 ± 264 | 44.9 |
| 20 | 5000 | 217 ± 60 | 27.9 | 612 ± 272 | 44.5 |

**Table S2:**

Mean [ln(*N_j_*)] of the cumulative number of fragments for a set of six tresses for untreated (UT) and bleached (BL) hair. The standard deviations (STD) and the Coefficients of Variation (CV) are given.Number of tresses = 6.

| **Inspection Count** | **Cycles** | **UT**  **ln(N_j_) ± STD** | **CV,%** | **Bleach**  **ln(N_j_) ± STD** | **CV, %** |
| --- | --- | --- | --- | --- | --- |
| 1 | 250 | 2.9 ± 0.23 | 7.9 | 3.5 ± 0.43 | 12.1 |
| 2 | 500 | 3.2 ± 0.24 | 7.4 | 4.2 ± 0.48 | 11.5 |
| 3 | 750 | 3.5 ± 0.26 | 7.5 | 4.6 ± 0.47 | 10.3 |
| 4 | 1000 | 3.8 ± 0.25 | 6.7 | 4.8 ± 0.53 | 11.0 |
| 5 | 1250 | 3.9 ± 0.28 | 7.1 | 5.0 ± 0.52 | 10.3 |
| 6 | 1500 | 4.1 ± 0.30 | 7.3 | 5.1 ± 0.52 | 10.2 |
| 7 | 1750 | 4.2 ± 0.31 | 7.3 | 5.3 ± 0.52 | 9.9 |
| 8 | 2000 | 4.3 ± 0.31 | 7.2 | 5.4 ± 0.54 | 10.0 |
| 9 | 2250 | 4.4 ± 0.31 | 7.0 | 5.5 ± 0.52 | 9.5 |
| 10 | 2500 | 4.5 ± 0.29 | 6.5 | 5.6 ± 0.51 | 9.1 |
| 11 | 2750 | 4.6 ± 0.28 | 6.1 | 5.7 ± 0.51 | 8.9 |
| 12 | 3000 | 4.7 ± 0.28 | 6.0 | 5.8 ± 0.51 | 8.8 |
| 13 | 3250 | 4.8 ± 0.27 | 5.5 | 5.9 ± 0.50 | 8.4 |
| 14 | 3500 | 5.0 ± 0.26 | 5.3 | 6.0 ± 0.49 | 8.2 |
| 15 | 3750 | 5.0 ± 0.26 | 5.2 | 6.1 ± 0.48 | 7.9 |
| 16 | 4000 | 5.1 ± 0.26 | 5.1 | 6.1 ± 0.48 | 7.8 |
| 17 | 4250 | 5.2 ± 0.26 | 5.1 | 6.2 ± 0.48 | 7.8 |
| 18 | 4500 | 5.2 ± 0.27 | 5.1 | 6.2 ± 0.48 | 7.7 |
| 19 | 4750 | 5.3 ± 0.27 | 5.1 | 6.3 ± 0.48 | 7.7 |
| 20 | 5000 | 5.3 ± 0.27 | 5.0 | 6.3 ± 0.48 | 7.5 |
